# Reduced Photosynthetic Efficiency of Tilia (*Tilia tomentosa*) Exposed to Radio Frequency Electromagnetic Field (RF-EMF)—JIP-Test Analysis

**DOI:** 10.3390/plants15121824

**Published:** 2026-06-12

**Authors:** Julian Keller, Uwe Geier

**Affiliations:** Forschungsring e.V., 64295 Darmstadt, Germany; keller@forschungsring.de

**Keywords:** *Tilia tomentosa*, photosynthesis, senescence, stress, radio frequency electromagnetic fields, F_V_/F_M_, RC/CS_0_

## Abstract

The growing use of wireless technology significantly increases the exposure of all living organisms to radiofrequency electromagnetic fields (RF-EMF). However, the physiological effects of RF-EMF on plants have not yet been sufficiently researched. In this study, we investigated the effects of RF-EMF radiation in the frequency ranges 1890–1900 MHz (DECT) and 2.4 GHz plus 5 GHz (Wi-Fi) on photosynthetic performance of Tilia plants (*Tilia tomentosa*). The recorded fast chlorophyll fluorescence transients were used to analyze the structure and function of PSII by the JIP-test. The analysis of the fluorescence of chlorophyll *a* showed that the RF-EMF interfered with the electron transport processes of photosynthesis. *Tilia* plants exposed to RF-EMF induced decrease in photosynthetic efficiency (F_V_/F_M_) and inactivation of part of PSII reaction centers (RC/CS_O_). Observations of leaf senescence and lifespan over a period of 102 days showed that RF-EMF-exposed *Tilia* plants exhibited accelerated aging.

## 1. Introduction

The increasing use of wireless telecommunications technologies in recent decades has led to a significant rise in exposure to artificially generated radiofrequency electromagnetic fields (RF-EMF). Natural electromagnetic fields—including cosmic microwaves, infrared, visible light, UV, and gamma radiation—differ significantly from anthropogenically generated RF-EMF in terms of intensity, frequency spectrum, and polarization patterns [1]. The high levels of RF-EMF exposure we are currently facing are unprecedented in the history of the Earth. It has been found that key factors such as frequency range, duration and power density have varying effects on plant growth and development [2]. While most previous studies have focused on animals and humans, comparatively little research has been conducted on plants. Like other organisms, plants are exposed to RF-EMF in their environment. Numerous studies suggest that RF-EMFs can have an adverse effect on plants under certain conditions [3]. In a previous study, we identified a previously overlooked effect of RF-EMF on lettuce stress responses. The experiments showed that, under greenhouse conditions, RF-EMF caused only minimal direct stress, but interfered with the plants’ natural responses to light stress, making them more susceptible to light stress under field conditions. The outdoor study showed a significant and systemic decrease in photosynthetic efficiency and accelerated flowering time compared to control groups. Gene expression analysis revealed that two stress-related genes, violaxanthin de-epoxidase (VDE) and zeaxanthin epoxidase (ZEP), were significantly down-regulated in RF-EMF-exposed plants [4]. In another study, our results suggested that RF-EMF exposure weakens drought-induced hormetic responses in lettuce plants, both in terms of response magnitude and extent [5]. Reviews from 2024 and 2021 identify the increase in reactive oxygen species (ROS) as a key mechanism by which RF-EMF affect plants. This can cause changes in gene expression and various metabolic processes, which in turn can impair growth and development [2,6]. Studies investigating the effects of RF-EMF on trees remain scarce, although the limited available evidence suggests potentially harmful effects [7,8,9]. The study [7] documented tree damage within the radiation field of cellular base stations. Furthermore, one-sided crown damage, beginning on the side facing the antenna, suggests a possible link between RF-EMF and tree damage. At the molecular level, RF-EMF exposure has been associated with changes in gene expression; for instance, in tomato (*Lycopersicon esculentum*), at least five stress-related genes are upregulated in response to RF-EMF exposure [10,11,12,13]. Exposure of duckweed (*Spirodela polyrhiza*) to RF-EMF leads to growth retardation and developmental abnormalities [14]. A similar RF-EMF-induced growth inhibition was also observed in mung beans (*Vigna radiata*), shrub roses (*Rosa hybrida*), and radishes (*Raphanus sativus*) [15,16,17]. In addition, the impact of RF-EMFs on various processes in plants, including photosynthesis, gene expression, protein production and secondary metabolism, has also been reported [18,19,20,21]. A 2016 meta-analysis showed that 152 (89.9%) of a total of 169 studies published between 1995 and 2016 found evidence of physiological changes resulting from RF-EMF, while 17 studies (10.1%) reported no effects [22]. The number of studies is still too small, and only a few plant species have been investigated. In addition, the RF-EMF frequencies, exposure durations, and intensities vary widely across studies, which makes direct comparison of their results difficult. It should also be noted that much of the research on RF-EMF effects on plants has been conducted under strictly controlled greenhouse conditions and, in most cases, involved short-term exposure. Long-term studies or studies conducted under field conditions are currently available only to a limited extent [6].

This study aims to investigate the effects of long-term, continuous RF-EMF exposure on the photosynthetic efficiency and onset of senescence in *Tilia tomentosa* trees in a realistic outdoor environment. In this context, analyzing fast fluorescence kinetics, particularly using the JIP-test, is a valuable approach for identifying and characterizing plant stress responses [23].

## 2. Results and Discussion

### 2.1. Continuous RF-EMF Exposure Resulted in Decrease in Individual JIP-Test Parameters

The function and structure of the photosynthetic apparatus were investigated using JIP-test analysis during prolonged exposure to RF-EMF. In recent years, the JIP-test has been widely applied to assess plant performance under stress conditions [24,25,26]. To evaluate the effects of RF-EMF exposure on the photochemical processes of photosynthesis, all JIP-test parameters were analyzed to identify which aspects of photosynthesis were affected. Parameters influenced by RF-EMF were selected based on the following criteria: (1) at least one statistically significant difference compared to the control plants over the exposure period, (2) a consistent direction of the observed effects, and (3) a moderate to strong Spearman’s rank correlation coefficient (ρ) between exposure duration and photosynthetic efficiency. The following five parameters fulfilling the selection criteria are presented here, while all remaining parameters are provided in the Supplementary Material.

- φ_PO_ (also referred to as F_V_/F_M_)—reflects maximal quantum yield of primary photochemical reaction [26].
- F_V_/F_O_—is a sensitive indicator of the functional state of PSII, responds strongly to structural or stress-induced alterations, and is proportional to the activity of the water-splitting complex [27,28].
- φ_DO_ (also referred to as F_O_/F_M_)—quantum yield of energy dissipation [29].
- ABS/RC—Absorbed energy flux in antenna chlorophylls per PSII reaction center; effective antenna size [30].
- RC/CS_O_—active reaction center density per cross-section, derived from the division of the absorption flux per cross-section (ABS/CS_O_, [31]) by ABS/RC.

For each measurement time point, a single treated/control ratio is calculated as:

$$Treated/control\;ratio=\frac{Mean\;value\;from\;all\;measurements\;ofexposed\;plants}{Mean\;value\;from\;all\;measurements\;of\;control\;plants}$$

**Figure 1:**
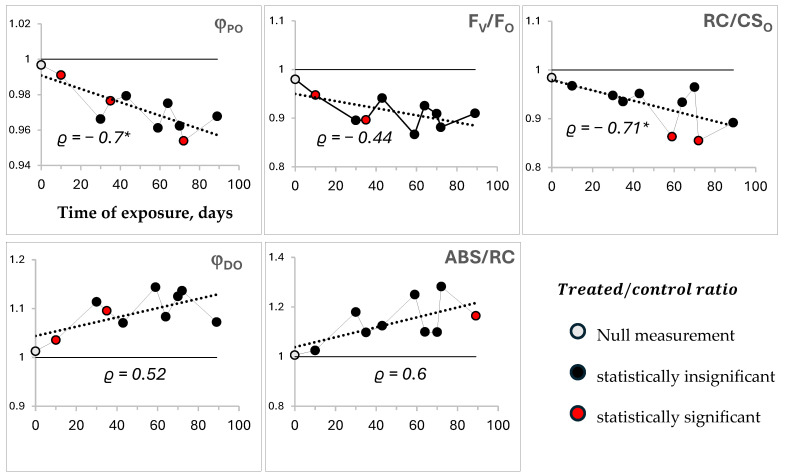
The course (Day 0 to Day 89) of the ratio between treated and control groups across the measurement time points for the OJIP parameters: φ_PO_ = (F_V_/F_M_), Fv/F_O_, φ_DO_ = (F_O_/F_M_), ABS/RC and RC/CS_O_. There are no statistically significant differences between treated and control groups in any null measurements (white dots). Black dots marked statistically insignificant differences between treated and control groups; red dots—statistically significant differences between treated and control groups. Also shown are Spearman’s rho rank correlation coefficients ϱ between the exposure time and the treated/control ratios. ϱ interpretation: −0.2 < ρ < 0.2: no correlation; −0.4 < ρ < −0.2 and 0.2 < ρ < 0.4: weak correlation; −0.6 < ρ < −0.4 and 0.4 < ρ < 0.6: moderate correlation; −1.0 < ρ < −0.6 and 0.6 < ρ < 1.0: strong correlation (as done in [4]). Asterisks (*) behind some ρ values indicate statistical significance of the Spearman correlation analysis (*p* < 0.05).

A treatment to control ratio of less than one is indicative of lower photosynthetic performance than controls, except for φ_Do_ and ABS/RC, where the opposite is true. Figure 1 shows the ratios between treatment and control throughout the exposure period and whether it is statistically significant. To calculate a correlation between exposure duration and photosynthesis efficiency, Spearman’s rho rank correlation coefficient ϱ was used, as previously done in [4]. Across all five JIP-test parameters, a consistent moderate-to-strong correlation was observed between exposure duration and the relative degradation of photosynthetic performance. This monotonic decline—analyzed via Spearman’s rho rank correlation coefficients—indicates that the impairment of the photochemical apparatus markedly intensified as the exposure period progressed.

The observed declining trends in several photosynthesis performance indicators follow the characteristic pattern of a typical plant stress response. φ_PO_ is one of the most stable JIP-test parameters, as it is rarely affected by growth conditions and only marginally affected by moderate stress [32,33,34,35]. A reduction in φ_PO_ typically suggests chronic stress damage rather than temporary acclimatization [36,37]. Plants often adapt to stress by deactivating PSII functional reaction centers and converting them into ‘heat sinks’ that dissipate excess energy. This protective mechanism directly reduces the number of active PSII reaction centers (RC/CS_O_) and leads to a corresponding decrease in F_V_/F_O_, as well as an increase in absorption per reaction center (ABS/RC) and the quantum yield of energy dissipation φ_DO_ [38,39,40,41].

### 2.2. Analysis of the Difference Curves at Certain Steps of the Induction Curve (IC) Shows That Photosynthetic Processes Are Influenced by Prolonged RF-EMF Exposure

Difference curves (DC) facilitate a detailed comparative analysis of the induction curve’s shape. By subtracting the fluorescence values of control plants from those of exposed plants, specific kinetic deviations become apparent. The emergence of positive or negative bands (i.e., localized kinetic deviations) in these curves reflects shifts in energy flux and overall photochemical performance, allowing for a precise evaluation of exposure-induced changes in photosynthetic efficiency [26,39,42].

Specifically, four primary bands characterize these deviations: L band (between O and 300 μs), K band (between O and J), H band (between J and I), and G band (between I and P) [42]. Among these, the L and K bands are particularly important as they serve as sensitive pre-symptomatic markers that appear even before any visible signs of stress become apparent [43].

We compared the induction kinetics of the treatment and control groups at the baseline (Day 0) and final (Day 89) measurement time points. At the baseline, no statistically significant differences were detected between the groups. By the final measurement, however, distinct deviations appeared: while no significant abnormalities were observed in the H- and G-bands, the L- and K-bands showed statistically significant positive shifts.

L-band

The part of the ICs between 50 and 300 µs is double normalized using W_OK_ = (F_t_ − F_o_)/(F_K_ − F_o_). Subtracting the IC values of the control plants from the RF-EMF-exposed plants (ΔW_OK_ = (W_OK_)_RF-EMF_ − (W_OK_)_control_) gives the L-band. The functional integrity of PSII —specifically antenna size and the energetic connectivity between reaction centers—is highly dependent on the protein packing density within the photosynthetic apparatus [44]. The L-band, in particular, reflects the structural arrangement or ‘grouping’ of PSII units within the thylakoid grana membrane, as well as the connectivity between the light-harvesting complexes (LHCII) and the reaction centers [39,44,45].

**Figure 2:**
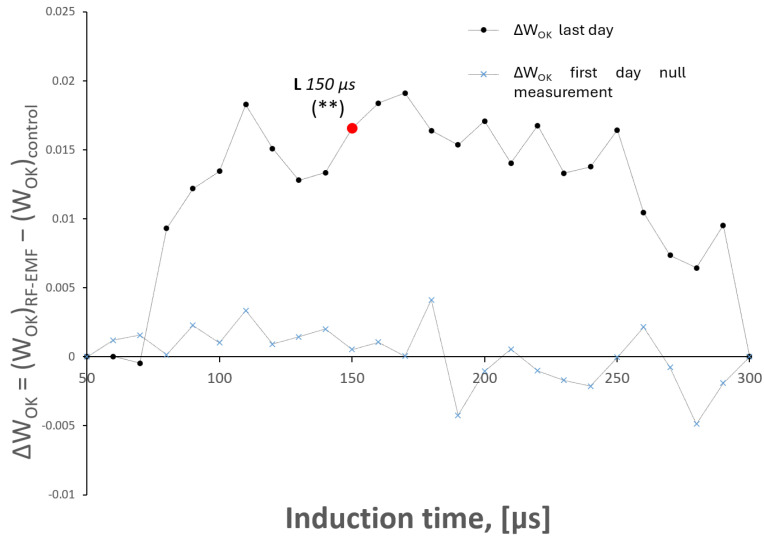
Difference kinetics (ΔW_OK_), calculated as ΔW_OK_ = (W_OK_)_RF-EMF_ − (W_OK_)_control_, illustrating the kinetic differences between RF-EMF-exposed and control samples. Each curve represents the difference between exposed and control plants at a specific time point (day 1: baseline measurement without exposure; day 89: final day of the experiment). Within the L-band, a red dot marks the characteristic point at approximately 150 µs. ** highly significant (*p* < 0.01) (Mann–Whitney U test).

The emergence of a positive L-band at 150 µs in this study (Figure 2) suggests a potential ungrouping of the antenna complexes as a result of long-term RF-EMF exposure [31].

K-band

In order to examine the ICs’ intercept between O (50 µs) and J (2 ms), the ICs are double-normalized using W_OJ_ = (F_t_ − F_O_)/(F_J_ − Fo)). DC was calculated using ΔW_OJ_ = (W_OJ_)_RF-EMF_ − (W_OJ_)_control_ and shows the K-band. The K-band is intrinsically linked to the stability of the oxygen-evolving complex (OEC) on the donor side of PSII. The emergence of a positive K-band typically reflects a kinetic imbalance where the rate of electron flow from the OEC to the reaction centers is insufficient to meet the demand of the acceptor side [39,46,47]. This phenomenon is generally induced by the partial inactivation of the OEC [30] and/or an increase in the functional PSII antenna [48].

**Figure 3:**
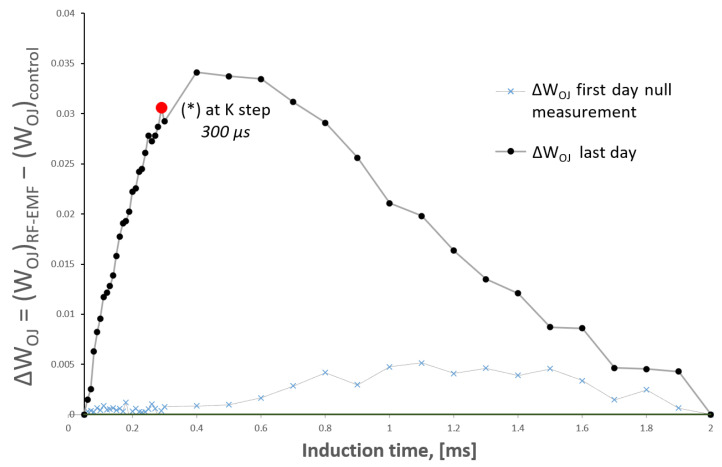
Difference kinetics (ΔW_OJ_), calculated as ΔW_OJ_ = (W_OJ_)_RF-EMF_ − (W_OJ_)_control_, illustrating the kinetic differences between RF-EMF-exposed and control samples. Each curve represents the difference between exposed and control plants at a specific time point (day 1: baseline measurement without exposure; day 89: final day of the experiment). Within the K-band, a red dot marks the characteristic point at approximately 300 µs. * Statistically significant (*p* < 0.05).

In our study, a statistically significant positive K-band was observed at 300 µs by the end of the exposure period. This was accompanied by a notably higher effective antenna size (ABS/RC) in the RF-EMF-exposed group (Section 2.1). These findings suggest that the increase in functional antenna size—driven by the deactivation of reaction centers—likely induced the aforementioned kinetic imbalance, manifesting as the positive K-band (Figure 3).

### 2.3. Analysis of Membrane and Leaf Models of Energy Fluxes Reveals That Photosynthetic Processes Are Affected by RF-EMF

We applied the Membrane and Leaf Models of Energy Fluxes at the final measurement time point (Day 89) to compare the RF-EMF-exposed and control plants. These models provide a graphical representation of the relationship between the energy fluxes of light energy absorption (ABS), excitation energy trapping (TR_O_) and unused energy dissipation (DI_O_), both on the basis of a single RC and per excited cross-section (CS_m_). It also provides the density of active (QA-reducing) RCs and the proportion of silent RCs. These silent, inactive RCs are not able to convert the light energy into photochemistry and emit this excitation energy as heat [30,48,49].

**Figure 4:**
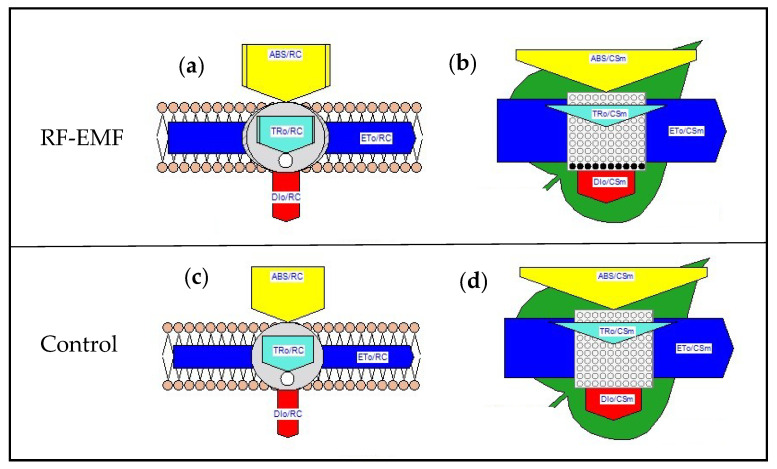
The membrane models on the left (**a**,**c**) show the specific energy fluxes per RC. The leaf models on the right (**b**,**d**) present the phenomenological energy fluxes per excited cross section CS_m_. The relative size of the individual fluxes is indicated by the width of the corresponding arrow. The oval around TR_O_/RC in the membrane model shows the density of active RCs (RC/CS_m_), while the hatched area around the oval of TR_O_/RC indicates the silent RCs. In the leaf model white circles indicate the active RCs (QA-reducing RCs) and black circles the inactive RCs, also called heat sinks or silent RCs [49]. These silent RCs are not able to convert the light energy into photochemistry and emit all this excitation energy as heat [48]. ABS/RC = light absorption flux per reaction center (apparent antenna size); trapped energy flux per reaction center; ET_O_/RC electron transport flux per reaction center; DI_O_/RC the dissipation energy per reaction center; Models are drawn with Biolyzer Software Version 4.0.30.03.02.

By the final measurement (Day 89), the RF-EMF-exposed plants displayed notably higher specific energy fluxes per reaction center, specifically for ABS/RC (*p* = 0.02), TR_O_/RC (*p* = 0.01) and DI_O_/RC (*p* = 0.05). Furthermore, the leaf models quantified a 10% proportion of silent RC (represented as black circles) in the exposed group (Figure 4).

### 2.4. Long-Term RF-EMF Exposure Led to Accelerated Senescence

**Figure 5:**
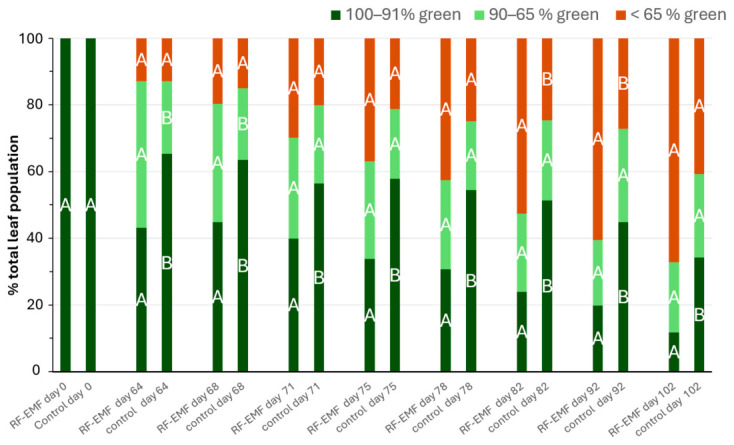
Relative distribution of leaf condition in RF-EMF-exposed and control plants. At the beginning of the observation period, all leaves were fully green (100%). Over time, this initial state was classified into three categories: dark green (91–100% green leaf area), light green (65–90%), and brown (<65%). The figure thus illustrates the temporal change in the proportion of each leaf condition category relative to the total leaf population. Different letters (A and B) indicate statistically significant differences between groups (*p* < 0.05).

To investigate the effect of continuous RF-EMF exposure on leaf senescence, the onset of senescence was monitored in both treated and control plants. RF-EMF-exposed plants exhibited earlier senescence, as indicated by a reduction in green leaf area and an increase in discolored leaf area. Figure 5 shows the percentage ratio of leaves that begin to discolor during the irradiation period. Continued irradiation resulted in a more rapid onset of senescence. The proportion of leaves with a 100–91% green area was significantly higher in the control group after prolonged irradiation. An earlier onset of senescence was also observed in *Trifolium arvense* plants exposed to RF-EMF [3].

## 3. Summary and Conclusions

Our in-depth JIP-test analysis reveals that long-term exposure to RF-EMF leads to a steady impairment of the photosynthetic apparatus. This is reflected in the declining trends in φ_PO_, F_V_/F_O_, RC/CS_O_ as well as the increasing trends of φ_Do_ and ABS/RC, in the treatment-to-control ratios over the course of the experiment. Furthermore, by the final measurement, the RF-EMF-exposed plants displayed higher energy fluxes per active reaction center, and 10% of the reaction centers were inactivated (converted into silent centers).

These tendencies are *phenomenologically* similar to the established plant protective mechanisms against photooxidative damage [40,50,51]. Such stress conditions often lead to the formation of Reactive Oxygen Species (ROS), which can cause direct photooxidative damage to the D1 protein and the manganese cluster of the oxygen-evolving complex (OEC). In this context, the conversion of active centers into ‘heat sinks’ helps to manage excess excitation energy and serves as a safeguard against the over-excitation and over-reduction in the electron transport chain [49,52,53,54].

The similarity between RF-EMF exposure and photooxidative stress is further supported by the senescence analysis, as the exposed plants exhibited significantly earlier leaf aging. It is well established that irreversible damage to the photosynthetic apparatus—driven by chronic photoinhibition and the accumulation of ROS—can shorten the functional lifespan of leaves [55,56].

In our previous study on lettuce, RF-EMF exposure was linked to the downregulation of VDE and ZEP—two enzymes essential to the xanthophyll cycle which safeguards plants against photooxidative damage. A disruption in this cycle compromises a plant’s ability to safely dissipate excess light energy, thereby increasing its vulnerability to photoinhibition. We previously discovered that light stress in combination with RF-EMF exposure led to a significant reduction in the maximum photochemical quantum yield F_V_/F_M_ of photosystem II and non-photochemical quenching (NPQ) compared to the control [4].

We postulate that long-term RF-EMF exposure also weakens the *Tilia tomentosa* response to photooxidative stress, ultimately inducing the observed stress-like symptoms. We recognize that this remains a hypothesis at this stage; given the nature of current literature and the inherent complexity of in situ environmental studies, this postulation is an interpretation of the physiological signatures rather than a definitive demonstration of causality. Furthermore, given that multiple abiotic stressors often produce overlapping physiological signatures, it cannot be ruled out that other external factors have contributed to or amplified these observed effects through synergistic interactions.

Nevertheless, the parallel between our findings and known photoinhibition pathways, combined with the potential for RF-EMF to interfere with fundamental protective mechanisms like the xanthophyll cycle, underscores an urgent need for targeted investigation. As wireless technologies and weather extremes continue to intensify, understanding whether RF-EMF functions as a stressor—either independently or by reducing plant resilience to other environmental pressures—is a critical area for future research. Understanding these effects is essential for ensuring the resilience of urban ecosystems in an increasingly digitized and climatically unstable world.

## 4. Material and Methods

### 4.1. Plant Cultivation and RF-EMF Exposure

The experiment was conducted at the Forschungsring e.V. experimental field in Darmstadt, Germany (49°49′57.4″ N, 8°34′22.2″ E). Young *Tilia tomentosa* trees were were cultivated in soil pots at the experimental field using F.-E. Typ SoMi Appel substrate (Hawita, Vechta, Germany), which already contained DCM ECO-Xtra1 fertilizer as supplied by the manufacturer.

The experiment was conducted during the summer-to-autumn transition, spanning from 28 June to 19 September 2022. Detailed time-course micrometeorological data are presented in the Supplementary Materials. At the beginning of the experiment, the 2-year-old plants were divided into two groups of 10 trees each. Trees were planted in separate pots. One group was exposed to RF-EMF while the other served as the control group. Control plants, placed adjacent to the RF-EMF-exposed area, were shielded from nearby RF-EMF emitters by a fine-mesh metal fence (mesh size: 13 mm squared holes; height: 120 cm) and served as the reference group. The RF-EMF-exposed plants were positioned around the RF-EMF-emitting devices at an equal distance of approximately 1.5 m to ensure uniform exposure. There were no microclimatic deviations between the RF-EMF-exposed group and the shielded control group. To ensure absolute parity in environmental conditions, both groups were positioned approximately 10 m apart in a flat, uniform, open field with minimal surrounding obstructions or structural shading. Furthermore, the metal mesh fence was not built as an enclosure around the control group; rather, it was installed as a single free-standing vertical lateral barrier positioned midway between the two groups.

Due to the lack of literature on the impact of high-frequency radiation on woody perennials, there was insufficient baseline data to determine which frequency would have the most significant physiological effect. In modern urban and suburban environments, vegetation is continuously exposed to a complex mixture of simultaneous ambient signals from sources such as DECT smart hubs and dual-band 2.4/5 GHz Wi-Fi networks. To evaluate how real-world signal mixtures affect urban canopy trees like *Tilia tomentosa*, we simulated a realistic, high-density anthropogenic RF-EMF scenario with frequency ranges of 1880–1900 MHz (DECT) and 2.4 and 5 GHz (Wi-Fi). RF-EMF exposure was provided by two Wi-Fi systems (FritzBox 7530; AVM, Berlin, Germany) in combination with a DECT base station and two DECT phones (Motorola t412+, Motorola, Chicago, IL, USA; eco-mode switched off). The DECT phones were maintained in continuous communication with an additional terminal. RF-EMF radiation levels were measured using two broadband RF analyzers (Gigahertz Solutions, Langenzenn, Germany). Peak power flux densities in the 1880–1900 MHz and 2.4 GHz range, measured with the HF59B analyzer (Gigahertz Solutions, Langenzenn, Germany) (700 MHz–2.7 GHz) on plants, were 800 µW/m^2^. In the 5 GHz Wi-Fi band, measured with the HFW35C analyzer (Gigahertz Solutions, Langenzenn, Germany) (2.4–6 GHz) also on plants, values of 400 µW/m^2^ were recorded. In contrast, RF-EMF measurements of both the ambient background and the shielded control plants yielded values < 8 µW/m^2^ for the 1880–1900 MHz/2.4 GHz range and <4 µW/m^2^ for the 5 GHz band, which is less than 1% of the recorded intensities and below the reliable quantification threshold of the measuring devices.

Fast chlorophyll fluorescence kinetics were measured and statistically analyzed after 48 h of acclimatization to ensure comparability between experimental groups. The RF-EMF emitters were then activated, and measurements were conducted at discrete intervals.

### 4.2. Measurements of Fast Chlorophyll Fluorescence Kinetics

Fast chlorophyll fluorescence kinetics of leaves were measured using a Pocket PEA device (Hansatech Instruments, King’s Lynn, UK). The measurement duration was 1 s, and a saturating light intensity of 3500 µmol m^−2^ s^−1^ was applied. Measurements were conducted at least 2 h after sunset, allowing for natural dark acclimation.

Prior to the experiment, leaves of the same age were pre-selected at random and marked for longitudinal monitoring (two on each tree). During each recording interval, a single measurement was performed per leaf. However, once visible signs of senescence appeared on the initial sample set (Day 43), the protocol was adjusted by transitioning all measurements to the younger leaves at the next nodal level across all plants to maintain physiological comparability.

JIP-test parameters were calculated using PEA Plus software (Version 1.13, Hansatech, King’s Lynn, UK). The results were averaged across all individual measurements from the same group.

### 4.3. Leaf Senescence Analysis

Leaf senescence was evaluated via visual phenotypic classification based on progressive leaf discoloration. At the onset of the experiment (Day 0), all leaves were verified as fully healthy and green, representing 100% green leaf area.

Throughout the exposure period, leaves were monitored dynamically and classified into three distinct physiological categories based on visual inspection of chromatic shifts and necrotic surface area:

- Dark green: 91–100% green leaf area (healthy leaves)
- Light green: 65–90% green leaf area (early chlorophyll degradation)
- Brown: <65% green leaf are (advanced senescence and irreversible tissue death).

Individual leaves were assessed according to these visual criteria and assigned to their respective categories.

### 4.4. Statistical Analysis

Due to the non-normal distribution of the data, the non-parametric Mann–Whitney U test was used to compare treated and control groups. Statistical significance was defined as *p* < 0.05. All statistical analyses were performed using Jamovi (Version 1.6.23).

The monotonic relationship—where variables tend to change in a consistent direction but not necessarily at a constant rate—between exposure duration and photosynthetic performance was assessed using Spearman’s rho rank correlation coefficients. Correlation analysis was performed in Microsoft Excel using the Real Statistics Resource Pack add-in.

## Data Availability

The original contributions presented in this study are included in the article/Supplementary Material. Further inquiries can be directed to the corresponding author.

## Supplementary Materials

The following supporting information can be downloaded at: https://www.mdpi.com/article/10.3390/plants15121824/s1. File S1: Comparisons of JIP-test parameters between RF-EMF-exposed and control Tilia tomentosa plants over 10 measurement timepoints; File S2: Radar plots showing the multi-parametric JIP-test profiles of *Tilia tomentosa* under RF-EMF exposure across 10 measurement timepoints; File S3: Meteorological conditions during the experimental period.
