# Supplementary material for: Reduced Photosynthetic Efficiency of Tilia (Tilia tomentosa) Exposed to Radio Frequency Electromagnetic Field (RF-EMF)—JIP-Test Analysis
Source: Plants (Basel). 2026 Jun 12;15(12):1824. doi: 10.3390/plants15121824 (PMC13306421; doi:10.3390/plants15121824)

# Reduced Photosynthetic Efficiency of Tilia (*Tilia tomentosa*) Exposed to Radio Frequency Electromagnetic Field (RF-EMF) – JIP-Test Analysis

File S1. **Comparisons of JIP-test parameters between RF-EMF-exposed and control Tilia tomentosa plants over 10 measurement timepoints**

Box plots illustrate the distribution of $RC/CS_O$, $F_V/F_M$, $ABS/RC$, $F_V/F_O$ and $F_O/F_M$ of RF-EMF-exposed (blue/unshaded) and control (red/shaded) *Tilia tomentosa* plants over 10 measurement timepoints (Day 0 to Day 89). Asterisks (*) indicate statistical significance at $\alpha$ = 0.05 (Mann–Whitney U test).

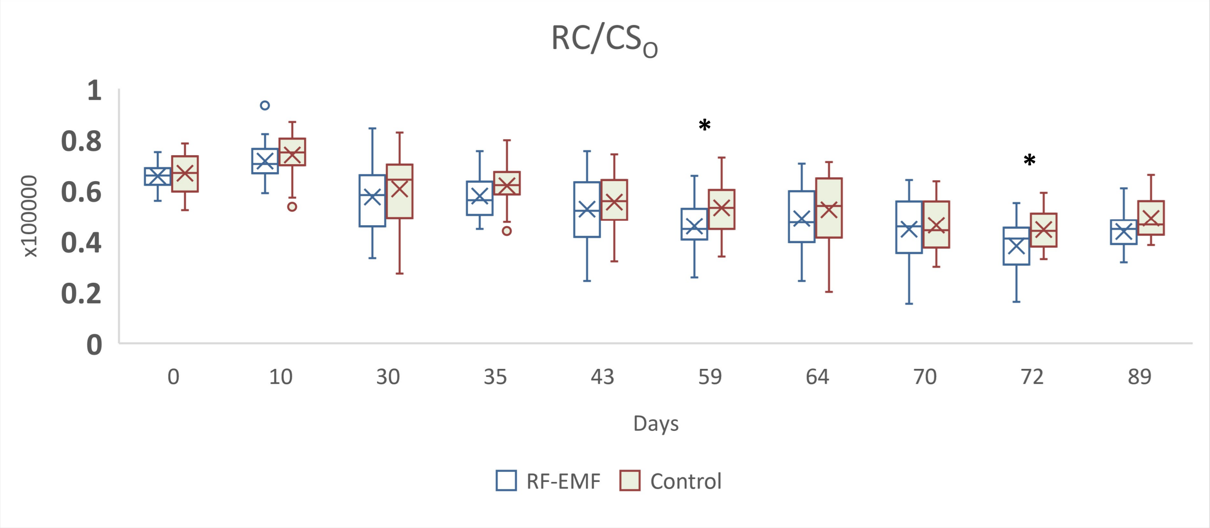


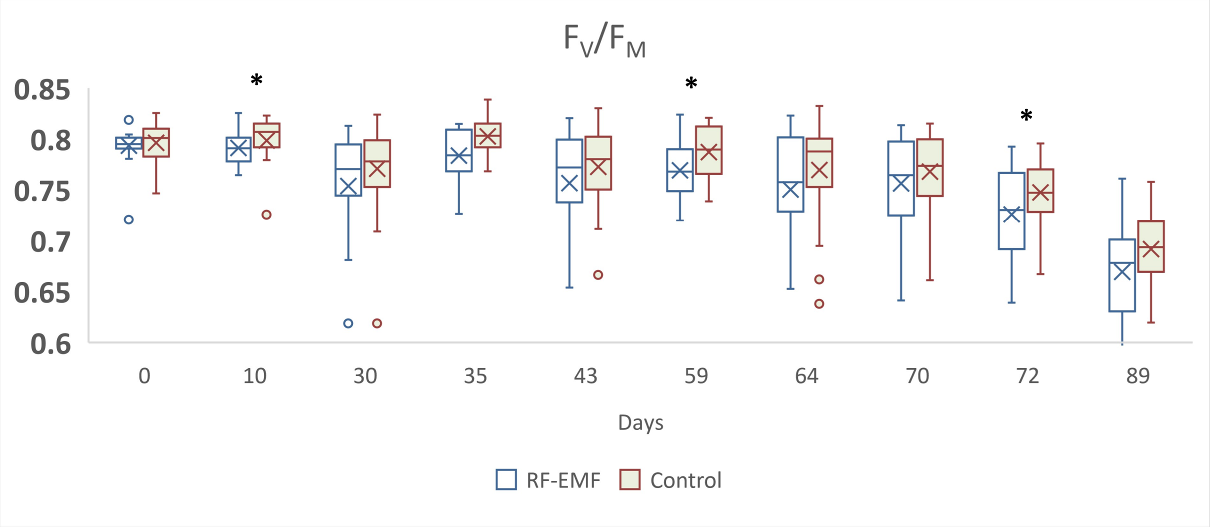

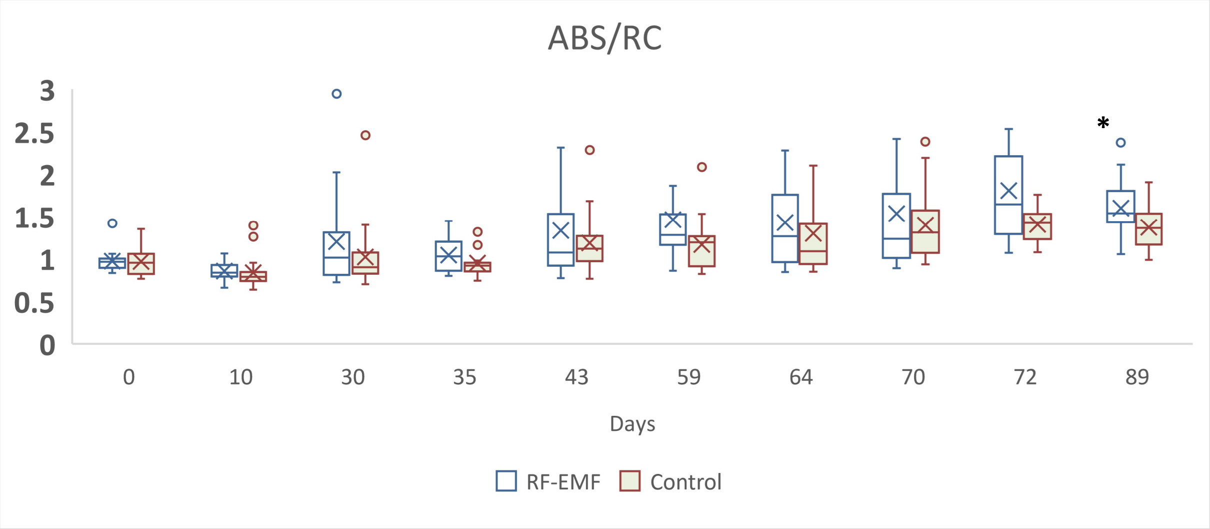
ABS/RC
3
2.5
2
1.5
1
0.5
0
*
0
10
30
35
43
59
64
70
72
89
Days
RF-EMF
Control

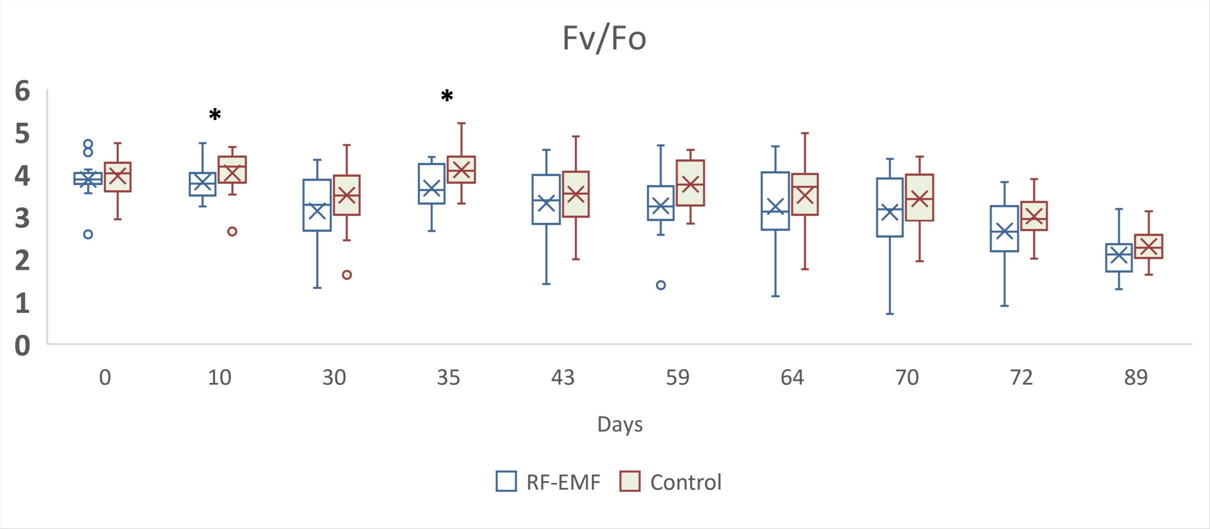
Fv/Fo
6
5
4
3
2
1
0
*
*
0
10
30
35
43
59
64
70
72
89
Days
RF-EMF
Control

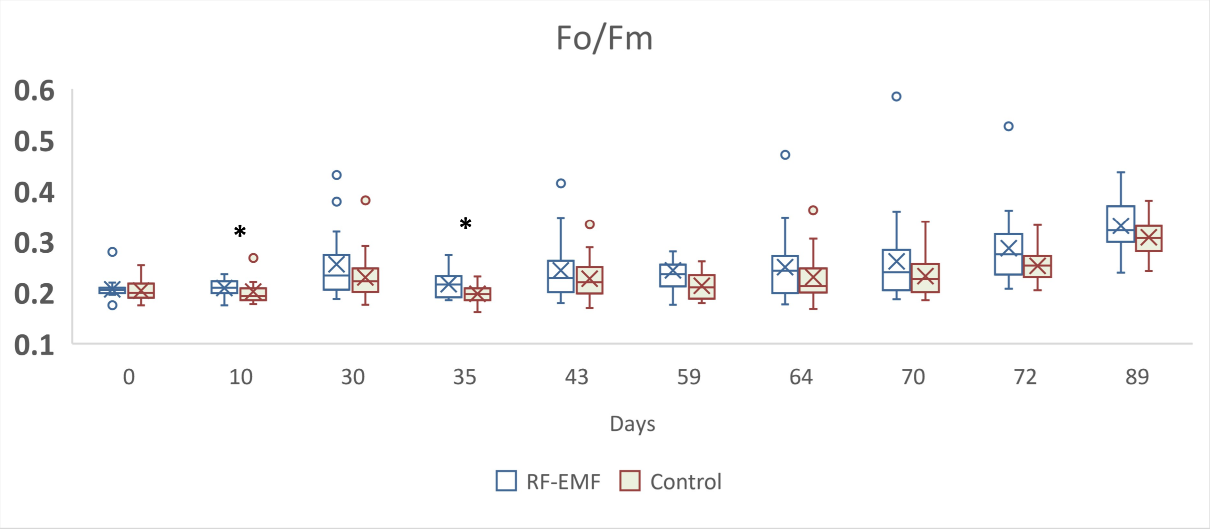
Fo/Fm
0.6
0.5
0.4
0.3
0.2
0.1
*
*
0
10
30
35
43
59
64
70
72
89
Days
RF-EMF
Control

**File S2. Radar plots showing the multi-parametric JIP-test profiles of *Tilia tomentosa* under RF-EMF exposure across 10 measurement timepoints.**

All data points for the RF-EMF-exposed group (orange) are normalized relative to the corresponding values of the control group (blue), which serves as the baseline (represented as a uniform, central reference polygon at 1). Deviations outside or inside this reference line illustrate the relative increase or decrease of each parameter in response to RF-EMF exposure. Asterisks (*) indicate statistical significance at $\alpha = 0.05$ (Mann–Whitney U test). Double asterisks (**) means $p < 0.01$.

Day 0

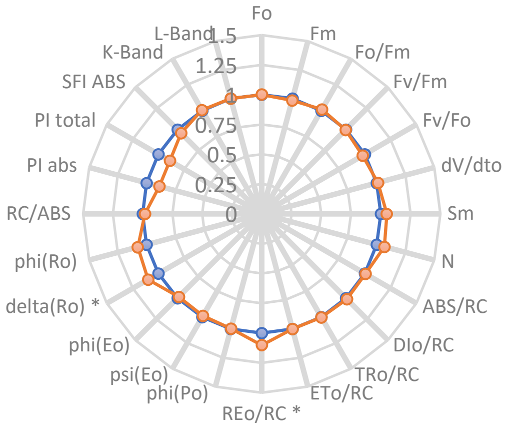


Day 10

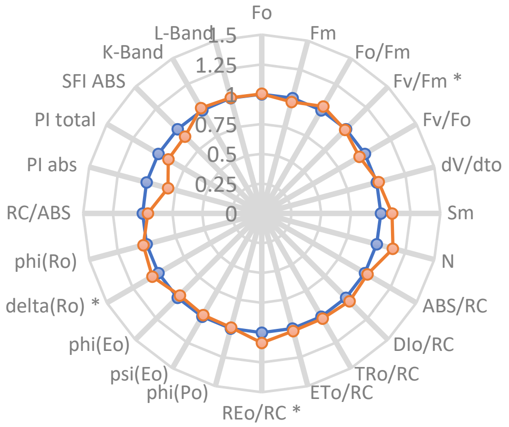


Day 30

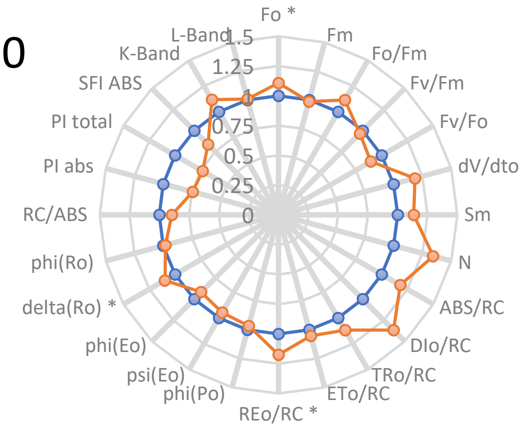

## Day 35

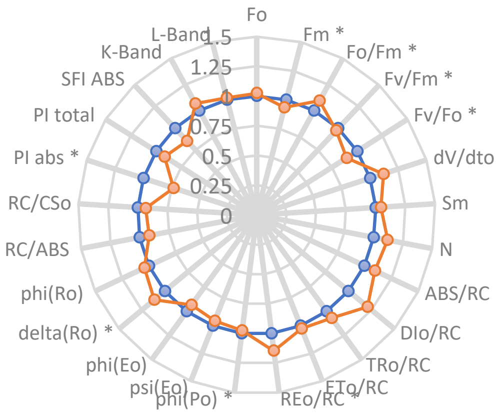


## Day 43

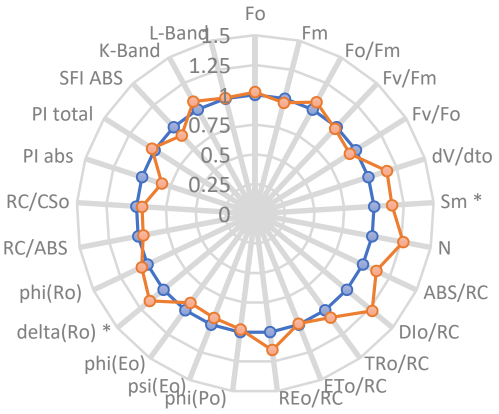


## Day 59

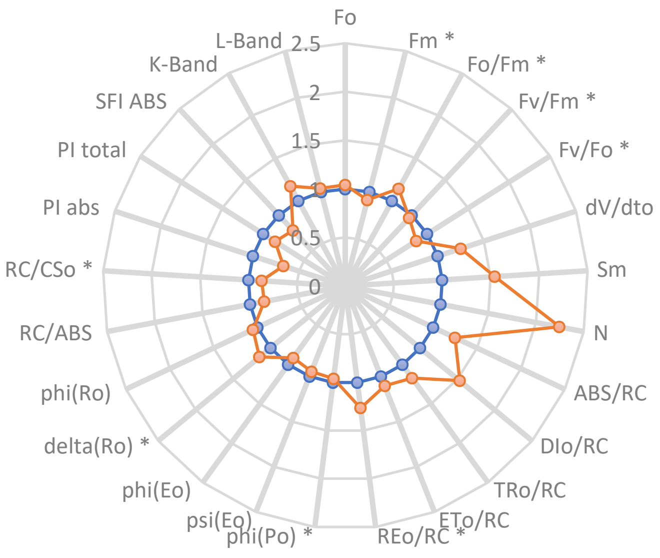


## Day 64

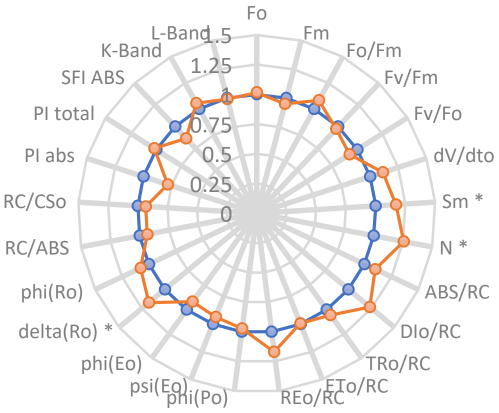

## Day 70

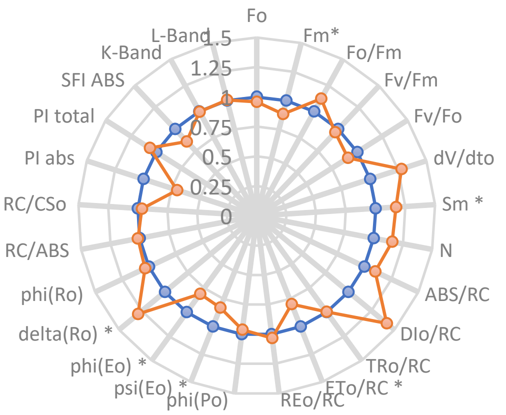


## Day 72

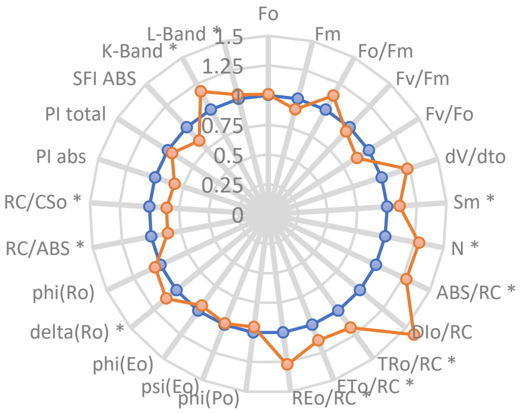


## Day 89

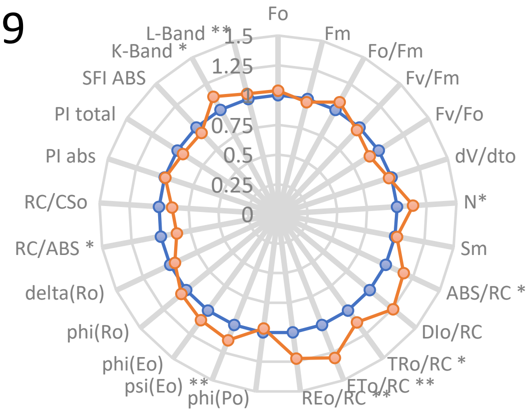

**File S3. Meteorological conditions during the experimental period**

The time-course plots illustrate the ambient environmental baseline for the experiment, capturing: 28

- Temperature: Daily maximum and minimum temperature ranges (°C)
- Rainfall: Total precipitation rates expressed in millimeters per hour (mm/h)
- Daily Light Integral (DLI): Total photosynthetic photon flux density received per day (mol/m².d)

The specific measurement timepoints are:

- Day 0 28.06.2022
- Day 10 08.07.2022
- Day 30 28.07.2022
- Day 35 02.08.2022
- Day 43 10.08.2022
- Day 59 26.08.2022
- Day 64 31.08.2022
- Day 70 06.09.2022
- Day 72 08.09.2022
- Day 89 19.09.2022

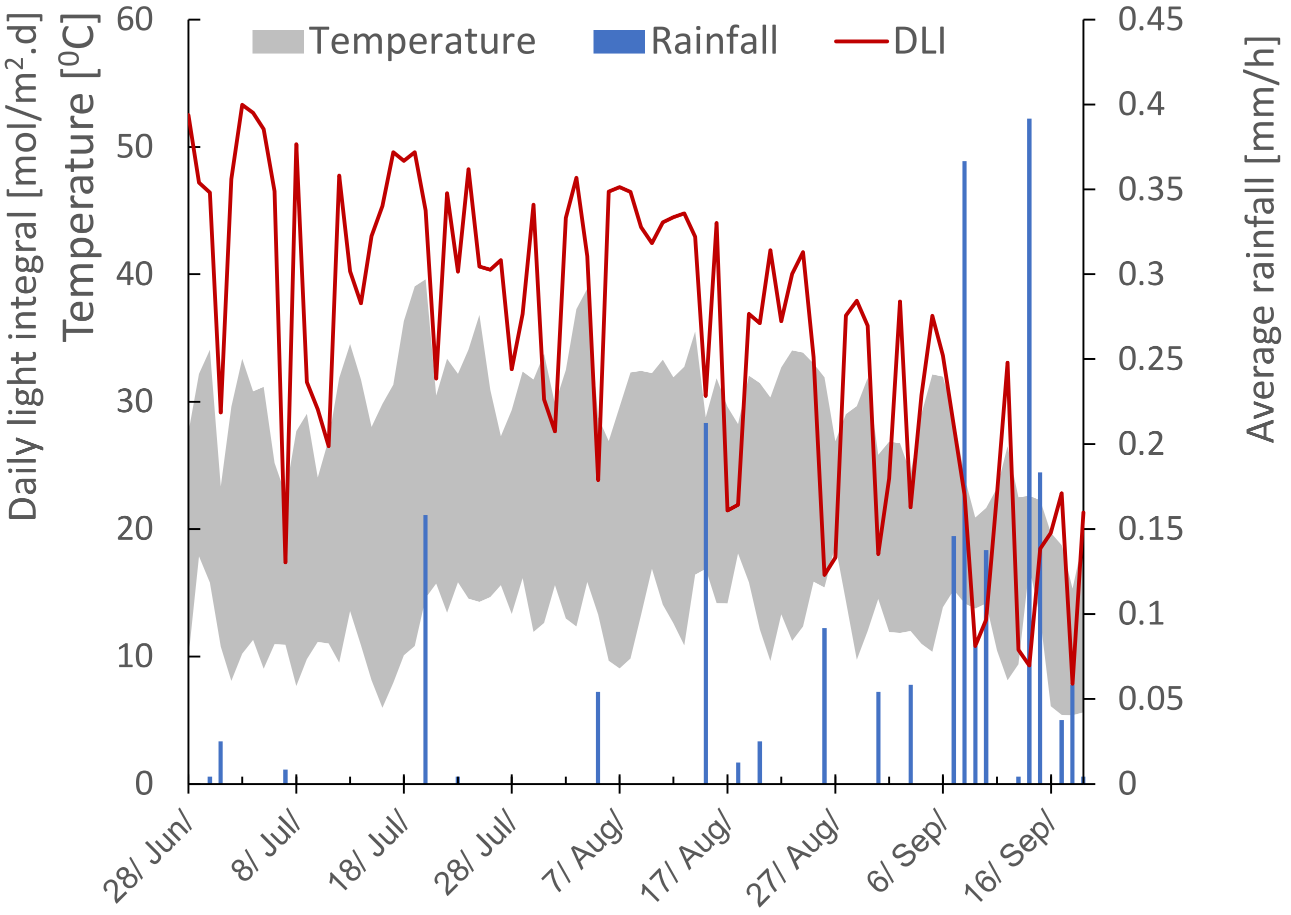

Supplement: Supplementary file 1 [file plants-15-01824-s001.zip › plants-4329600-supplementary.pdf]
